# Microbiome Associated with Olive Cultivation: A Review

**DOI:** 10.3390/plants12040897

**Published:** 2023-02-16

**Authors:** Rogério Melloni, Elke J. B. N. Cardoso

**Affiliations:** 1 Institute of Natural Research, Federal University of Itajubá (Unifei), Itajubá 37500-903, MG, Brazil; 2 Luiz de Queiroz College of Agriculture, University of São Paulo (Esalq/USP), Piracicaba 13418-260, SP, Brazil

**Keywords:** *Olea europaea*, management, mycorrhiza, rhizobacteria

## Abstract

International research has devoted much effort to the study of the impacts caused to the soil by different management practices applied to olive cultivation. Such management involves techniques considered conventional, including the control of spontaneous plants with herbicides or machines, inorganic fertilizers, and pesticides to control pests and diseases. Equally, some producers use sustainable techniques, including drastic pruning, the use of cultivars that are tolerant to diseases and adverse climates, the use of organic conditioners in the soil, the maintenance of vegetation cover with spontaneous plants, and the use of inoculants, among others. In both conventional and sustainable/organic management, the effects on soil quality, crop development, and production are accessed through the presence, activity, and/or behavior of microorganisms, microbial groups, and their processes in the soil and/or directly in the crop itself, such as endophytes and epiphytes. Thus, our present review seeks to assemble research information, not only regarding the role of microorganisms on growth and development of the olive tree (*Olea europaea* L.). We looked mainly for reviews that reveal the impacts of different management practices applied in countries that produce olive oil and olives, which can serve as a basis and inspiration for Brazilian studies on the subject.

## 1. Introduction

The olive tree (*Olea europaea* L.) has a prominent position in the world food market due to the high demand for products such as olives and olive oil. Pieces of research addressing biological factors in the soil-plant relationship are increasing in focus for the improvement of cultivation, from the seedling stage to field production, especially research related to the impact of management on soil microbiota and soil functions. Another important point is the use of microbial groups, such as arbuscular mycorrhizal fungi (AMF), and plant growth-promoting rhizobacteria (PGPR).

Since the largest producers of olives and olive oil in the world are in the Mediterranean, the vast majority of work with such studies has been concentrated in these regions, generating a range of information that is often diffuse and contradictory. There is much information presented in this review, which refers to studies published mainly in Spain, Portugal, Italy, France, Greece, Tunisia, and Morocco. In opposition to those, in Brazil, there is a significant lack of information about the soil microbiota and/or the microbiome of the olive tree, both under controlled conditions of seedling production and those carried out in situ in mature orchards under production. Although olive cultivation was introduced in Brazil around the 19th century, in some regions with favorable environmental conditions such as the South and Southeast, cultivation success is moving slowly. This drawback is mainly due to the lack or insufficiency of studies related to the nutritional/climatic requirements of the culture (and its cultivars) and equally to some factors or management that directly affect its productivity. Hence, due to the low commercial production, the country still needs to import a large part of its olive oil from traditional producing countries. However, studies have emphasized the excellent quality of some national olive oils, as described in the review of Filoda et al. [1], which discusses the chemical and sensorial quality of Brazilian olive oils and how these aspects influence the identity of the Brazilian product.

The olive tree is a plant that reacts differently to management conditions. Thus, it is highly mycotrophic, forming associations with AMF and reacting with wide variations to the inoculation of different fungal species. Olive groves naturally colonized by native fungi are found commonly around the world [2,3]. Comprehending this relationship and communication between plants and fungi, as well as between colonized plants, is a massive challenge currently detailed in a recent paper by Boyno and Demir [4]. In this research, the authors reveal the substances involved and the uncertainties present in these communications, emphasizing the intelligent connection network between the symbionts, increasing the interest of researchers in studying and evaluating the health and behavior of plants, such as olive trees, when interacting with microorganisms.

Besides the bipartite association observed between fungi and the olive tree, or vice-versa, other associations were recorded and studied, sometimes forming a tripartite system with rhizosphere bacteria of diverse characteristics, or even with endophytic bacteria and fungi. In this regard, several studies seek to understand and apply this knowledge to cultivation practices in a sustainable way, especially when olive tree cultivation occurs in places with evident abiotic stresses, including high or low temperature, high or low moisture, poor and/or degraded soils, and biotic factors related to the occurrence of pests and diseases.

Therefore, reviews have been addressing the effects of the application of microorganisms or microbial groups on olive cultivation, both in Brazil [5] and worldwide [6]. However, along with the incessant search for sustainable processes of olive tree management and production, there is a need to collect updated information, encouraging researchers worldwide to engage in new reviews, such as this one, which aims to systematize information published in the form of scientific papers, especially in the last 10 years (2012–2022). There are reviews that relate the effect(s) of different management methods applied in olive cultivation, which work with microorganisms either in the soil or associated directly with the plant.

Therefore, the research results of our present review are presented in the form of three major topics, namely: management affecting edaphic conditions and soil microorganisms, relationships between microorganisms and disease control in olive trees, and microbiology of olive trees in Brazil and future perspectives.

## 2. Methodology

The methodology used consists of conducting a systematic review, starting with a question, followed by the sampling of existing data and systematization regarding the subject matter under discussion [7]. Specifically, we suggest the following research questions: Which are the main topics of recent published papers in scientific journals dealing with microbiology or soil microorganisms applied to olive cultivation? Which studies report the effect of cultivation methods on the soil microbiota and its processes? Which studies aim to understand and enhance the role of the microbiome on olive tree growth, development, and production? Is it possible to systematize the main information from these papers, to enable management recommendations and future perspectives of its application in olive cultivation in Brazil and worldwide?

The protocol consisted of a search for studies in two platforms, Capes Periodicals Portal (https://www-periodicos-capes-gov-br.ezl.periodicos.capes.gov.br/; accessed on 7 October 2022) and Scielo Brazil (https://www.scielo.br/; accessed on 7 October 2022), selecting only studies published as scientific articles in national and international journals during the ten last years. In the Capes Periodicals Portal, we used the keywords “soil microbiology” and “Olea europaea”, selecting articles related to agriculture and agronomy, and eliminating articles about the application of microorganisms in industrial processes, focused on olive oil quality. In the Scielo Brazil platform, we used the keywords “*Olea europaea*” and the olive tree (in the title), filtering for the fields of Agricultural and Biological Sciences, with no limitation of year and language.

Thus, we selected articles published primarily between 2012 and 2022, focusing on the subjects related to the olive tree (*Olea europaea* L.): management, microorganisms, fungi, mycorrhiza, bacteria, microbial activity, microbial biomass, disease control, biotic stresses, abiotic stresses, endophytes, and microbial interactions. We divided the analyses resulting from those studies and will present them in three main topics: management affecting edaphic conditions and soil microorganisms, relationship between microorganisms and disease control in olive trees, and microbiology of olive trees in Brazil and Future perspectives.

## 3. Results

### 3.1. Management Affecting Edaphic Conditions and Soil Microorganisms

The management applied during cultivation affects directly the soil, its characteristics, and properties, reflecting on the microbial communities. Several microorganisms or microbial groups and their processes are used as indicators of soil quality due to their sensitivity. In function of these studies, we can affirm that two types of management stand out: sustainable and conventional management systems. In each of them, there were variations in the application of pruning methods, application of conditioners derived from industrial residues of olive oil production (pressing residue and biochar), variation of irrigation systems, methods of soil cover control, and inoculation of microorganisms combined or applied individually. Next, we are going to detail the studies related to the effect of these management systems on soil properties and soil microorganisms.

**Figure 1:**
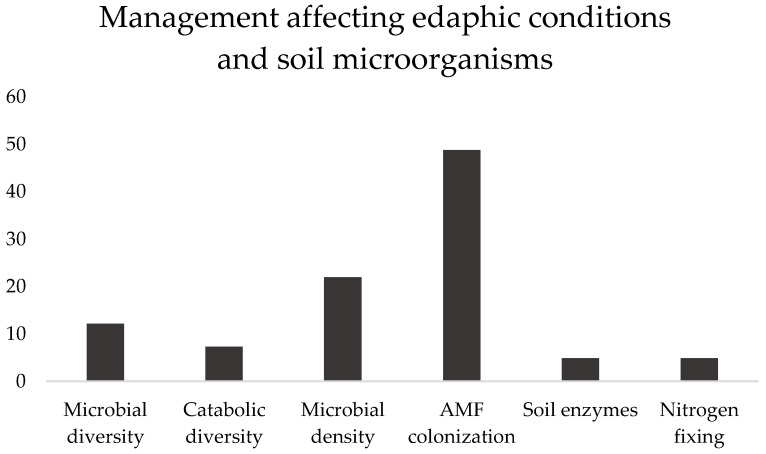
Subjects covered in the selected papers, in percentage, addressing the effect of management with different soil microorganisms, and in different edaphic conditions, between brackets. Microbial diversity [8,10,12,17,26], Catabolic diversity [10,11,27], Microbial density [9,15,17,19,22,23,25,28,37], AMF colonization [13,23,29,30,31,32,33,34,37,38,39,40,41,42,43,44,45,46,48,49], Soil enzymes [14,17], N_2_ fixing [19,21].

The articles selected in this subsection addressed six subjects, namely microbial diversity, catabolic diversity, microbial density, soil enzymes and N_2_ fixing (Figure 1), and AMF colonization (the vast majority), which will be presented and discussed.

Overall, sustainable soil management in orchards can cause positive effects on the soil, favoring productivity through increased microbial biomass, activity, and complexity. Comparing both systems, Sofo et al. [8] investigated the effect of sustainable versus conventional management, for twelve years. The sustainable method consisted of “no-till, self-seeding weeds—mainly Graminiae and Leguminosae, and mulch derived from olive tree prunings”. The conventional method consisted of “frequent tillage, including severe pruning with residues removed from the orchard”, for 12 years. Then they compared soil microbial composition and metabolic diversity in a Maiatica cultivar olive orchard in southern Italy (Ferrandina, Italy; 40°29′ N, 16°28′ E). Through cultivation and molecular methods, the authors found a higher occurrence of fungal and bacterial groups (mainly Streptomyces) in soil under sustainable practices and higher metabolic diversity by Biolog and enzyme activities. Thus, they confirmed the positive effect of sustainable methods on the soil microbiota and, directly, on soil functionality, primarily through the periodic application of organic matter.

Given the Mediterranean conditions (high temperature and low moisture), soil management should prioritize the increase of organic carbon and microbiological diversity. Five years later, the same authors evaluated the effect of different management techniques, conventional versus sustainable, during 18 years in a mature olive grove (*Olea europaea* L., cv. ‘Maiatica’, 70 years old). This was also in the Mediterranean (again in Ferrandina, southern Italy, in a semi-arid climate with an average annual precipitation of 565 mm [9]), presenting this average from 1995 to 2015. The conventional system involved weed control, pruning and removing branches from the areas, and NPK mineral fertilization, while the sustainable system involved light pruning and row disposal of the residues on the ground, maintenance of spontaneous plants on the ground, drip irrigation, and reduced nitrogen fertilization applied once a year. The sustainable system promoted an increase in organic matter in the superior layers of soil (C and N), a reduction in pH values, and an increase in the density of bacteria related to the N cycle, with no effect on total bacterial activity and diversity. The authors concluded their study by emphasizing the importance of maintaining the soil alive and with a biodiversity as high as possible to preserve its quality over time.

In another comparative study between 41 soils under organic cultivation and 49 soils under conventional olive tree cultivation in Andalusia, southern Spain, Montes-Borrego et al. [10] measured the effect of these practices on soil chemical and biological properties. Through traditional methods, cultivation analysis, Biolog and enzyme activities, combined with multivariate analysis, the authors observed that, overall, organic cultivation showed higher values of organic matter, organic C and N, C/N ratio, as well as higher microbial diversity and catabolic capacity to utilize different organic substrates. This was the first demonstration of soil health maintenance in commercial organic olive orchards in southern Spain, encouraging other producers to promote and conserve soil quality under olive cultivation in the region.

To get an idea of the practices commonly applied in olive groves in Lebanon, Boukhdoud et al. [11] analyzed the effect of no-till, joint cultivation with vetch (*Vicia sativa* L. var. sativa—Fabaceae), as well as conventional cultivation methods. The soils presented varying chemical and biological characteristics in different geographical positions (coastal area, 34°21′38″ N, 35°44′31″ E, and inland area, 34°18′35.2″ N, 35°48′27.8″ E). Differences were observed in the chemical properties of coastal soils (lower N and C alkyl fraction) and microbiological properties (higher index of catabolic diversity), regardless of agricultural practices. The negative effect of conventional management on soil microbial function was observed only regarding location (higher on the coast), but with reduced effect when cultivating with Vicia. This study revealed an effect of the geographical position in which the olive crop is planted on soil properties, at the coast, bigger C mineralization and changes in the microbial catabolic profile, particularly when conventional management is applied in olive cultivation. Thus, the authors emphasize that conventional management is not adequate for introduction in the Mediterranean coastal area.

A different paper, which compares different management effects on soil microorganisms, was presented by Caliz et al. [12]. The authors used molecular methods (16S rRNA gene studies), changes in the composition and abundance of ammonium oxidizers, bacteria and nitrite oxidizers in the rhizosphere of 96 olive groves under different climatic conditions, management practices, soil properties, and olive cultivars (mainly ‘Lechin’, ‘Manzanillo’, ‘Nevadillo,’ and ‘Picual’). Most bacteria of the genera *Nitrososphaera*, *Nitrosospira tenuis*, and *Nitrospira* sp. modulated their effect according to soil properties, such as pH and texture, and to the olive cultivar. However, in the present paper, there were detected only minor effects due to the olive grove management and soil cover practices, olive grove age, or organic matter content on the microbial groups evaluated.

Land cover management in olive groves is a challenge for productivity, soil degradation, and oil quality. To investigate land cover in olive groves, García-González et al. [13] assembled an experiment, also in Spain (40°4′21″ N, 3°31′11″ W), under field conditions, in an olive grove cv. Cornicabra, in the Mediterranean region, with a semi-arid climate, varying the ground cover treatments. The cover varied between annual Bitter vetch Willd. Fabaceae, perennial *Brachypodium distachyon* L.P.Beauv., Poaceae, and spontaneous plant species mostly of the family Brassicaceae, and no cover (conventional control). The use of annual plants increased AMF colonization by 50% when compared to the treatment without cover. The cover removal also affected the quality of the oil, reducing the ripeness and polyphenol index. Interestingly, the authors associated the observed AMF colonization (71.5% in the annual crop, 50.3% in the perennial crop, and 3% in the spontaneous vegetation), along with changes in soil nutrients such as Cu, B, and other elements, with the quality of the oil produced. Although the removal of vegetative cover increases the productivity of olive trees via the reduction of competition for water and nutrients, the introduction of vegetative cover promoted an increase in polyphenol content, ameliorating the oil quality, while protecting the soil from erosive processes, and is recommended as part of the olive cultivation management process.

Regarding the use of the sustainable management on olive cultivation, Moreno et al. [14] evaluated the effect of techniques related to vegetative cover and chemical weed control through biological indicators of soil quality, i.e., size and structure of the bacterial community by PCR-DGGE. The activity of six enzymes (dehydrogenase, o-diphenoloxidase, β-glucosidase, phosphatase, urease, and arylsulfatase), representing the cycles of C, P, N, and S, were also investigated. Corroborating data previously presented, only minor changes were found. Ground cover promoted greater bacterial biomass and functional microbial diversity than bare soil. However, eliminating spontaneous plants with herbicides reduced functional diversity. Thus, based on the sensitivity of the biological indicators studied, the authors concluded that maintaining covers on soils would be the best option for managed olive groves, especially in regions with water scarcity.

As we observed, herbicides can alter the structure and function of soil via direct effects on various components of its microbiota. Usually, this effect is reported for herbaceous and annual plants, and seldom for forest and woody perennial species, such as olive trees. In this sense, Bórtoli et al. [15], in the province of Cordoba, Argentina, evaluated the short-term effect of glyphosate (Roundup UltraMax, Monsanto Co, St. Louis, MO, USA) on soil quality and microbial diversity in soils with olive trees in two different cases: previous pesticide use and another with no such application history. In the latter situation, the effect of the application was more prominent, with a significant imbalance and increase in the density of culturable Gram-negative bacteria, evaluated based on the fatty acid profile, despite the recommended dose not differing from the control. Thus, the authors once again confirmed the negative effect of herbicide application or any other spontaneous plant control method on the soil microbiota and its processes. Olive grove management with the maintenance of soil cover with spontaneous plants has also contributed to an increase in the carbon stock in the soil, according to another study conducted in Brazil by Guimarães et al. [16], which directly implies positive effects on physical, chemical, and biological attributes due to the increase in organic matter content.

This elevation in organic matter content, apart from the addition of organic conditioners, which we will address further on, was also found when abandoning or ceasing activities in the olive grove. The abandonment of olive groves is a significant phenomenon associated with economic and social factors, which must receive researchers’ attention. On this focus, Palese et al. [17] analyzed the effect of abandonment during 25 years on the chemical, biochemical, and microbiological properties of soil from olive groves located in the rural area of Lucera (southeastern Italy—41°27′38.32” N, 15°22′13.75” E), comparing it with an adjacent reference area olive grove with extensive minimal cultivation practices and no fertilization. The abandoned soil presented higher organic matter content, total N, and pH due to the non-cultivation and natural input of material with high C/N, and a higher number of cellulolytic bacteria and β-glucosidase activity, an indicator of a more evolved stage of the soil. However, it showed lower numbers of total bacteria and fungi, and lower microbial diversity, as assessed by Biolog, as the result of a specialization related to the low quality of the organic substrates added to the soil, reinforcing the effect of different management systems applied to olive cultivation.

Another crop management technique widely used in olive cultivation is the pruning system. In this regard, Malamidou et al. [18] investigated the effects of the decomposition of olive tree residues (leaves and twigs) under organic management on soil properties in northern Greece (40°29′ N, 22°55′ E) with the cultivar Chondrolia Chalkidikis. The authors noted that the mass loss and release of C, N, K, and Mg were higher in the leaf residues compared to those from the twigs, indicating that the incorporation of these residues into the soil contributes to enrichment with N, K, Ca, and Mg, but the incorporation of additional fertilizers is still needed. Thus, management of the incorporation of pruning residues remains relevant in olive cultivation, aiming to increase organic matter and nutrient cycling from mineralization processes by soil microorganisms.

In the municipality of Sutamarchán, latitude 5°37′ north and longitude 73°34′ west, which belongs to the province of Alto Ricaurte, Colombia, Bello Alvarez et al. [19] evaluated the density and activity of diazotrophs in the rhizosphere of young olive trees under four years old. The trees were submitted to pruning and free growth, and elements such as N, NH_4_^+^, NO_3_^−^, organic C, P, Fe, and Mg were added, which act directly on the biological nitrogen fixation process (BNF), relying on nitrogenase. The olive cultivar studied here is not well known, and is planted only since 1950 in this region. The results indicated that the pruning process generated soil acidity and reduced leaf and soil N levels, resulting in a high density of inefficient diazotrophs with low nitrogenase activity, possibly because they are free-living. The authors concluded that such activity hardly contributes to the direct N requirements of the crop but is limited to impacts on microbial metabolism and soil functionality.

Few studies were found to evaluate the possibilities of using concentrated organic fertilizers and biostimulants to increase the growth or production of olive trees. Thus, utilizing organic soil conditioners as a biostimulant in the growth promotion of young olive trees in Italy, Mazeh et al. [20] tested the effect, under controlled conditions (pots with cv. Leccino) and in the field (cv. Moraiolo), of mineral fertilizers versus organic conditioners. This fact reinforces the use of organic conditioners as agents of biostimulation in young olive orchards, which should be included in the management of this crop. Regarding the organic matter, the use of organic residues in olive cultivation has been encouraged as a sustainable management strategy. Many studies verified that such use caused improvements in the quality of the soil and its microbiota. Among these residues, we can highlight those from the post-pressing of the olives during olive oil production, the olive mill wastewater (OMW), and the biochar produced from the combustion of the dry residue formed at 350 °C and 500 °C. The first of those materials was used in many of the conducted studies, mainly in the Mediterranean. There the soil conditions are acidic (pH 5.1, moderate electrical conductivity of 9.1 dS m^−1^), with significant concentrations of NPK (1340 mg L^−1^, 720 mg L^−1^, and 6200 mg L^−1^, respectively), a dark coloration, and great potential for utilization despite the high content of phenols and chemical oxygen demand (COD).

In 1992, the use of alpechin (“wastewaters from olive oil mills”) in soil was introduced as a fertilizer. However, several doubts remained, especially regarding the impacts on soil microorganisms and their activity, such as that of nitrogenase in N_2_ fixers. Garcia-Barrionuevo et al. [21], in Spain, described the effect of this residue on the nitrogenase activity in agricultural soils under aerobic and controlled conditions. The authors noted that the residue could affect the growth of N-fixers and the N-fixation process, but *Azotobacter* spp. tolerated high concentrations (15–20% *w*/*w*), still with a stimulatory effect on their activities, even considering that the residue added phenolic acids to the soil (considered toxic to plants). The authors emphasized that the application of the diluted residue appears not to affect the free-living N-fixers in the soil, which would be involved in saving and supplying N to the olive groves.

To evaluate the short-term effect of adding this residue to topsoil layers under olive groves in the city of Ouled Jaballah (Tunisia), Mechri et al. [22] employed a methodology based on “ester-linked fatty acid methyl ester (EL-FAME)” to analyze the structure of the affected microbial community. This method allowed the observation of variations in the community, with specific groups favored, mainly with a reduction in Gram-negative bacteria and an increase in unsaturated fatty acids (common in Gram-negative bacteria and fungi). The fungus/bacteria connection increased with the use of the residue, and these results were the first to report changes in the FAME profile in soils that received olive residue application when cultivating the crop.

One year later, at the same location (North, latitude 35°12′, East, longitude, 10°59′), Mechri et al. [23] analyzed the effect of different amounts of this residue (30, 60, 100, and 150 m^3^ ha^−1^) on photosynthesis, root soluble carbohydrates, root colonization by mycorrhiza, soil properties, and microbial community structure (with emphasis on AMF) in olive groves. The authors used the “soil fatty acid methyl ester (FAME)” methodology to quantify fungal biomass and roots, and they observed an increase in organic C, C/N, exchangeable P, and K after one year of residue use, with no change in pH. They also observed increased saprophytic fungi but reduced AMF colonization, photosynthetic rate, and total dose of root-soluble carbohydrates. The authors indicated that there was a possible competition between saprophytic fungi and AMF in the use of root carbohydrates, reducing root colonization by the second group. This was the first report concerning the alteration of AMF when applying residues in doses above 30 m^3^ ha^−1^, and should be considered in further studies when recommending its use in the management of olive cultivation.

A review by Ntougias et al. [24] discussed the first 20 years of the application of this residue in olive cultivation. After using the residue, the authors observed the dominance of members of Alpha-proteobacteria, Beta-proteobacteria, Gamma-proteobacteria, Firmicutes, and Actinobacteria, containing many diazotrophs, depending on the cultivar. A further concern of the authors, and not covered in the studies presented earlier, was the detection of fecal bacteria and other human and plant pathogens. Therefore, this opens new possibilities for research involving these microorganisms and the capacity of indigenous microorganisms to promote their inactivation or destruction.

In the same year, Nasini et al. [25] evaluated the effect of adding “Solid olive-mill waste (SOMW)” in solid and liquid fractions of the olive pressing process and olive oil production on soil cultivated with olive tree cv. Leccino, in central Italy, near Assisi (12°56′ E longitude, 43°11′ N latitude). The authors noted that organic C did not increase in the soil due to the microorganisms’ rapid utilization of the organic matter. However, they observed an increase in exchangeable K, with no inhibitory effect on the microbiota, despite a low increase in viable bacteria, bacterial spores, and fungi, with positive effects on the vegetative growth of olive trees and fruit production without compromising the sensory characteristics of olive oil. These authors recommended using those residues in olive groves, when possible.

Proietti et al. [26] also studied the same residue generated in the decanter and its derived compost in olive grove cv. Leccino, also in central Italy, near Assisi (12°56′ E longitude, 43°11′ N latitude), analyzing the effect on soil chemical characteristics, bacterial abundance and community structure, vegetative growth, oil production, and quality. After three years of use, the top layer did not increase in total organic carbon, in contrast to the observations for available P and exchangeable K. Concerning microbiological aspects, no difference was found in viable bacteria and biodiversity by PCR-DGGE when compared to the control treatment (no use of the residue). However, due to the improvement in plant growth and the positive effect on oil quality, the results revealed the potential of using this residue in the soil, contributing to reducing fertilizer use in the olive crop.

The effect of agronomic practices that are usually applied to olive trees, such as residue addition and glyphosate herbicide, on the soil microbial community was studied by Boukhdoud et al. [27] in Alpilles, near the city of Marseille (southeastern France). They observed significant changes in the catabolic profiles of cultivable microbial communities and higher lipase activity in the soil after 2 months of glyphosate application, leading to a reduction in functional diversity. The authors concluded that adding residue along with glyphosate avoided the negative effect of the herbicide and, therefore, should be an indicated measure to maintain soil functionality when applying the herbicide in conventional olive cultivation management.

The long-term effects of using olive oil residue on the chemical properties of the soil, microbial community, and growth parameters are still not completely clear. Regni et al. [28] evaluated the effects one and two years after the use of this residue at two different depths (0–20 and 20–40 cm) on the soil chemical characteristics and soil microbial communities in a mature olive grove, 30 years old, with cv. Frantoio, close to Perugia, central Italy, at 12°59′ E longitude, 42°92′ N latitude. The authors found variations in chemical attributes (pH, salinity, available P, water-soluble organic C) within 14 days after application, returning to normality after 2 years, except for available P. Firmicutes, Proteobacteria, and Actinobacteria were the most abundant phyla, and, after 14 days, the residue had a negative effect only on Firmicutes. This suggests that only Gram-positive bacteria showed a more negative effect than the other phyla when applying this type of residue. This evidence suggests that the long-term use of residue does not affect the endemic soil bacterial community under olive trees, as well as photosynthesis activity, productivity, and fruit characteristics. Therefore, this result confirms the positive effects of residue use in olive cultivation, disregarding possible negative effects of long-term use, although further studies with this approach still are recommended.

Besides the olive trees, all plants constantly interact with microorganisms, with heavy interference from the management used. These microorganisms include arbuscular mycorrhizal fungi (AMF) and soil bacteria that provide ecosystem services related to vegetative growth, nutrition, and quality parameters. A general review of current research involving AMF inoculation, combined or not with rhizobacteria growth promoters in plant production, has been produced by Noceto et al. [29]. The authors emphasize the need to know the regulatory mechanisms that act in plant-symbiont and symbiont-symbiont interactions to develop agricultural practices and study the interactions, aiming at optimizing the symbiotic potential of plant root-associated microorganisms.

With regard to the other organic residue derived from the industrial process of olive oil production, biochar, Vejvodová et al. [30] studied the potential of its use. Along with the inoculation of AMF (Funneliformis mosseae—Glomus mosseae), the researchers set up a controlled experiment with wheat (*Medicago sativa* L.) as a test plant in contaminated soils with different concentrations of heavy metals (As, Cd, Pb, and Zn), in Granada (Spain). The results revealed an increase in K concentration and a reduction in soluble Cd in the soil treated with biochar, having an ambiguous effect on the mobility of As, Pb, and Zn. At the same time, inoculation of AMF did not affect the biochar effect on the soil, and the authors concluded that more research is necessary to expand the use of biochar, especially long-term research on contaminated soils. This is another knowledge gap, and it should be developed and recommended to find better management practices applicable to olive cultivation.

As we have noticed, soil conditioners and beneficial microorganisms are important agents that can increase the sustainability of olive tree agroecosystems. However, the effects depend on several conditions that require extensive research. Lopes et al. [31] conducted a 4-year monitoring experiment in an olive grove with cv. Cobrançosa, non-irrigated and mature (about 30 years old) in Mirandela (coordinates 41.513946 and −7.187348), in northeastern Portugal. They evaluated the effect of biochar, zeolites, basic NPK + B fertilization (50 kg ha^−1^ of N, P_2_O_5_, and K_2_O and 2 kg ha^−1^ of B) and commercial AMF inoculum on photosynthetic performance, nutritional status, olive-tree growth, and soil properties. Basic fertilization contributed over 21% to the olive tree’s growth, compared to the control treatment, mainly due to the N gain in the plants. Overall, the treatments promote no differences in photosynthetic rate, but it was significantly higher than the control. The use of biochar increased organic matter and CTC, stimulating biological activity without increasing the production of the olive trees. The authors found no effect of AMF inoculum (containing propagules of five different species—*Rhizophagus irregularis*, *Funneliformis mosseae*, *F. geosporum*, *F. coronatum* and *Claroideoglomus claroideum*). According to the authors, this failure may be due to the application of the inoculum in mature orchards, which are possibly already colonized by native fungi and more adapted to the local ecological conditions. Thus, the interest in using commercial AMF inoculum in mature olive orchards appears to be low in experimental conditions. According to the results of several studies, the use of AMF in the olive crop is conditioned to the seedling production phase which, when inoculated, promotes improvements, diminishing the stresses of transplanting, growth, and production.

The inoculation of beneficial microorganisms is a technique for the sustainable management of olive cultivation. The effects of AMF inoculation on olive trees are diverse and well-established. Inoculating olive tree seedlings cv. Cornicabra with Glomus mosseae, Glomus intraradices, or Glomus claroideum, Porras-Soriano et al. [32] observed, in Spain, in addition to better resistance to transplanting, a higher ability to absorb N, P, and K, both in saline (cultivation with added NaCl) and non-saline conditions. G. mosseae was the most efficient species in the reduction of the effects of salinity. This response is attributed to the higher absorption of K (which plays an important role in osmoregulation processes and tolerance to salinity), with high values in the growth of the aboveground tissues (163%) and roots (295%) under non-saline conditions, and of 239% (aboveground) and 468% (roots) under saline conditions.

Thus, commercial AMF inoculants were used during the preparation of olive seedlings in southern Spain to improve establishment in the field. However, further research is necessary to understand the effect of agricultural practices and the environment on the biodiversity of these fungi. Using crop-independent molecular methods, Montes-Borrego et al. [33] analyzed the AMF communities associated with 90 olive groves in southern Spain, 49 in conventional and 41 in organic systems, and the established edaphic effects. The authors noted that fungal distribution correlated strongly with some soil attributes, the agronomic characteristics of the cultivar, and the age of the plantation and irrigation regime, but not the management system and vegetation cover for erosion protection. Among the soil attributes, the effects of pH, texture, nutrient content, average evapotranspiration, rainfall, and minimum temperature were prominent. Molecular analyses revealed the presence of AMF of five families, with *Archaeospora* spp., *Diversispora* spp. and *Paraglomus* spp. Being the first recorded species on olive trees. The authors found a few isolates from the Claroideoglomeraceae and Glomeraceae families, not belonging to any species used as an inoculant in propagation and seedling preparation. The authors suggest that AMF may exert high host specificity, more than previously thought, influencing studies of inoculum production and the specific effects of the soil and environmental conditions where the olive groves are established.

In Argentina, Bompadre et al. [34] evaluated the effect of *Rhizophagus irregularis* (*Glomus intraradices*) inoculation on reducing transplantation stress of olive seedlings (*Olea europaea* L. cv. Manzanillo). The authors concluded that the inoculated plants increased growth and were protected against oxidative stress. Previously, this protective effect of host stress to drought, with increased vegetative growth and concentration of antioxidant enzymes, proline, P, Ca^2+^, and K^+^, was observed by Moreno-Galván et al. [35] when inoculating corn with Bacillus sp. The authors evidenced the complex mechanism of interaction, suggesting that the response of the plant to the inoculation is due to the specific correlation observed between plant-bacteria and changes in antioxidant enzymes. A similar effect was also observed by Filgueiras et al. [36], when red rice (*Oryza sativa* L.) was inoculated with growth-promoting bacteria of the species *Gluconacetobacter diazotrophicus*. These authors verified that the positive drought tolerance response was due to increasing the osmoprotectant solutes and antioxidant enzyme activity levels that protect against cell and plant damage. Thus, this important biotechnological methodology must be included and recommended for producing more vigorous seedlings, despite the higher initial cost.

The influence of inoculation of the AMF *Glomus intraradices* on microbial communities and sugar concentration was studied in the rhizosphere of olive tree cv. Meski, Tunisia, by Mechri et al. [37], using analyses of phospholipids and “neutral and lipid fatty acids” (PLFA and NLFA, respectively). Microscopic observations revealed that the extraradicular mycelium of the fungus showed the formation of branched absorbing structures in the rhizosphere of the olive tree, causing changes in the bacterial community, especially Actinobacteria, compared to non-inoculation. The authors detected higher concentrations of glucose and trehalose and lower concentrations of fructose, galactose, sucrose, raffinose, and mannitol in the rhizosphere with AMF, possibly as a consequence of changes in the environmental conditions in the rhizosphere provided by the AMF.

In another study conducted in Macedonia (northern Greece) under controlled conditions involving AMF and cultivars Koroneiki, Kothreiki, and Chondrolia Chalkidikis, Chatzistathis et al. [38] observed that AMF colonization varied among cultivars and soil conditions, along with root system morphology. In particular, *Gigaspora* sp. colonized the roots of all three cultivars, Glomus sp. only colonized Koroneiki, and maximum colonization occurred in Chondrolia Chalkidikis, also with higher aerial/root dry matter relation. The authors also observed variations in leaf concentrations of Mn, Fe, Zn, Ca, Mg, K, and P, associating the absorption efficiency with the AMF involved.

Briccoli et al. [39] conducted another study in Italy involving nutrient absorption under high concentrations of Mn in soil under greenhouse conditions, with the Italian olive tree cultivars Ascolana tenera, Nocellara del Belice, and Carolea, inoculated or not inoculated with AMF (commercial vs. native). Colonization promoted higher P absorption (generally double the concentration when compared to non-inoculation) and reduced Mn concentration (from 83% to 43%), mainly for native AMF, due to their adaptation to the experimental soil. There are few studies related to Mn in olive nutrition, although Paskovic et al. [40] evaluated the effect of foliar and soil (Zeolite) Mn on AMF colonization of seedlings of cv. Leccino, in Croatia. They concluded that foliar application of Mn, at a dose of 70 mg kg^−1^, favored the establishment of symbiosis, in contrast to the effect observed at higher Mn concentrations.

The management of irrigation systems also got the attention of researchers, especially in regions subject to moisture stress, such as the Mediterranean. In this regard, the additive effect of microorganism inoculation, especially AMF, to alleviate abiotic stresses due to effects on aggregation, water and nutrient absorption, and plant health are considered.

Thus, an important role of AMF in the soil is their contribution to soil aggregation, with direct consequences on aeration, water infiltration, and reduction of erosive processes. Kohler et al. [41] studied the involvement of three AMF species in aggregate formation and stabilization in soils with olive (*Olea europaea* L. subsp. sylvestris) in two areas of the semi-arid Mediterranean: Mazarron (37°31′7.8″ N, 1°27′58.7″ W) and Rellano (38°12′50.8″ N, 1°13′30.9″ W), both in the province of Murcia, Spain. In controlled conditions, the authors used the hypha roots separation compartment method to compare the effect of *Rhizophagus irregularis*, *Septoglomus deserticola*, and *Gigaspora gigantea* species on the structural stability of the hyphosphere and mycorrhizosphere in two soils in the region. Only *R. irregularis* significantly increased the percentage of stable aggregates in both soils, with a significant effect on the hyphosphere (stability higher than 30%, when compared to the uninoculated soil, and with 81% more hypha than the uninoculated), proving the importance of AMF in aggregate formation in poorly structured soils.

Under controlled conditions in Tunisia, Ouledali et al. [42] investigated the contribution of AMF to the resilience of olive trees in dry weather. Thus, olive plants of the cv. Zarrazi were inoculated with the commercial product Symbivit, which contained propagules of six different AMF species: *Glomus etunicatum*, *Glomus microaggregatum*, *Glomus intraradices*, *Glomus claroideum*, *Glomus mosseae*, and *Glomus geosporum*, before conducting growth in a drought regime for 40 days. The results highlighted that AMF-colonized plants were less affected by drought than those without mycorrhizae, and also presented higher turgor potential and stomatal function during the entire period of the experiment. Furthermore, the authors found higher nutrient absorption (K, N, Zn, and Fe) in the colonized plants which survived after the test, as opposed to those observed for the non-inoculated plants. In addition, when irrigation restarted, the colonized plants were able to re-establish themselves and recover from the stresses caused by the severe drought applied.

In another study aiming to prove the beneficial effect of AMF on the growth of olive tree cv. Picual subjected to abiotic drought stresses, Calvo-Polanco et al. [43] evaluated different species and water regimes under controlled conditions in southern Spain (37°31′43″ N, 2°54′34″ W). Native fungi collected from two regions were inoculated in soils from the same regions: semi-arid and humid, which were subjected to drought regimes. Results showed that plants grew better in soil from the humid region inoculated with fungi from the same region, as well as in soils with higher fungal diversity. However, the highest root hydraulic conductivity was obtained in plants inoculated with fungi of the humid region, but in the soil of the semiarid region, under moisture stress, revealing that such fungi could also act in differentiated regions.

To evaluate the efficiency of two commercial inoculants on the growth of young olive trees planted in acidic soils, Lopes et al. [44] conducted a field experiment in Mirandela (coordinates 41.451879 and −7.241243), northeastern Portugal, with olive tree cv. Cobrançosa. The tested inoculants were: (1) five AMF (*Rhizophagus irregularis*, *Funneliformis mosseae*, *F. geosporum*, *F. coronatum*, and *Claroideoglomus claroideum*), (2) nine AMF (seven of the genus Glomus, and two of the species Rhizophagus irregularis and Paraglomus brasilianum. Overall, the authors concluded that AMF increased the growth of young olive trees, in a direct association with photosynthetic activity and leaf area dimensions, and with no effect on the leaf concentration of nutrients such as N, P, K, and B. Such positive effects of AMF inoculation are important in olive crops to minimize the effects of stress from lack of water and imbalances of Ca and Mg in the plant tissues.

Specifically, when the irrigation of olive trees uses reclaimed water, which is common in areas with limited natural sources, problems are noticed due to the high salt levels (Na^+^ and Cl^−^), particularly those related to vegetative development. However, the application of AMF can minimize this effect and provide an efficient solution. With this aim, Ben Hassena et al. [45] evaluated the ability of AMF inoculants (*Glomus deserticola* and/or *Gigaspora margarita*) to act on the development of young olive trees (*Olea europaea* L. cv. Chetoui) when submitted to long-term water reclaimed in Tunisia (34°43′ N, 10°41′ E). The authors noted that plants irrigated with reclaimed water showed a significant reduction in water content, fresh matter and dry matter, gas exchange parameters, and chlorophyll and starch content when compared to using tap water, due to an increase in Na^+^ and Cl^−^, proline, soluble sugars, total polyphenols, and flavonoids. However, colonization with the different AMF, particularly in the 1:1 mixture of species, reduced the negative effects of reclaimed water, reducing Na^+^ and Cl^−^ concentrations and improving the other vegetative parameters already mentioned. This occurred possibly because the AMF-colonized plants have higher concentrations of proline and soluble sugars, and higher levels of antioxidants in their defense systems than the non-inoculated plants.

Ben Hassena et al. [46], under controlled conditions in Tunisia, evaluated the effect of AMF inoculation (*Glomus deserticola*, *Gigaspora margarita*, and a combination of *G. deserticola* and *G. margarita*) on young olive plants cv. Chetoui submitted to irrigation with saline residue. The authors observed an increase in electrical conductivity, and an accumulation of Na and Cl (12% and 133% in the leaves, and 9%8 and 106% in the roots, respectively) in plants that received the residue in comparison with plants without it, and an equal increase in antioxidant enzyme activities and H_2_O_2_ content. However, colonized plants showed lower Na and Cl content and higher macro and micronutrient content, especially when inoculated with *G. deserticola* and *G. margarita* combined (24% and 43% in leaves and 30% and 39% in roots, respectively), when compared to non-inoculated plants.

As mentioned earlier, the use of biostimulants in olive cultivation was used to mitigate the negative effects of high soil temperature and low soil moisture. In this regard, Graziani et al. [47] evaluated the effect of six biostimulants under controlled greenhouse conditions, in Napoli (Italy), using vegetative and ecophysiological parameters of olive tree cv. Salella. The biostimulants covered products based on Trichoderma, microalgae, seaweed, betaine glycine, kaolin, and the organic compound di-1-p-menthene (C_20_H_34_). The authors concluded that biostimulants based on betaine glycine and microalgae showed proven efficiency in protecting plants against the imposed abiotic stresses, resulting in increased stomatal conductance and water content. Kaolin promoted higher polyphenol and antioxidant activity in the leaves, revealing the high potential of the evaluated biostimulants for promoting the growth of young olive trees submitted to the evaluated abiotic stresses. However, the authors emphasized that further studies are required to test the efficiency of these biostimulants under field conditions over time, until the young trees produce olives.

The effect of *Rhizophagus irregularis*, when inoculated in a young olive tree cv. Picholine marocaine, combined with different irrigation systems under controlled conditions, was tested by Aganchich et al. [48] in Morocco. The lack of water during crop management negatively affected all the variables studied: stomatal conductance, leaf water content, vegetative growth, proline content, and sugar content. However, inoculation generally acted significantly on these growth variables compared to non-inoculated plants, even under water-limited conditions. In this regard, the authors concluded that AMF-colonized olive trees could tolerate water stress better, saving up to 50% of the irrigation water.

A summary of the papers on inoculation of microorganisms that interfered in processes related to olive tree growth under different conditions, mainly in drought conditions, is presented in Table 1.

Due to global warming, the growing range of many crops is changing to higher altitudes and latitudes, exposing plants to new climatic and environmental conditions. In this context, innovative technologies such as biostimulants and the manipulation of plant-bound microbiota are emerging. In northern Italy, Chialva et al. [49] studied olive groves rapidly expanding into cooler internal regions. For this purpose, they surveyed the prokaryotic and fungal microbiota in the endosphere of a cold-resistant and cold-sensitive cultivar (Leccino and Frantoio, respectively) during spring and winter. The results showed that the root microbiota of olive trees older than 20 years is stable, with low variations across seasons and genotypes. However, the authors found different abundance patterns for AMF and associated endobacteria, revealing the presence of intimate tripartite interactions. The authors suggested that a healthy and stable microbiota contributes to helping the olive tree in new environments and new climatic conditions.

Although the olive tree is considered well adapted to Mediterranean conditions, with climate change the climate projections indicate that the region will become hotter and drier. This will cause new challenges for olive growers, who will have to adjust their adaptation strategies in the short and long term. Specifically, the main impacts of climate change on olive cultivation in the Mediterranean and possible adaptation strategies can be found in a review conducted by Fraga et al. [50].

### 3.2. Relation between Microorganisms and Disease Control in Olive Tree

Tolerance or resistance to pests and plant diseases can derive from the host’s genetic condition or through processes/mechanisms initiated via rhizospheric, endophytic, and/or epiphytic groups of microorganisms. Specifically, the protective effect of AMF on the control of plant pathogens has been well-reported in plants, including olive trees.

**Figure 2:**
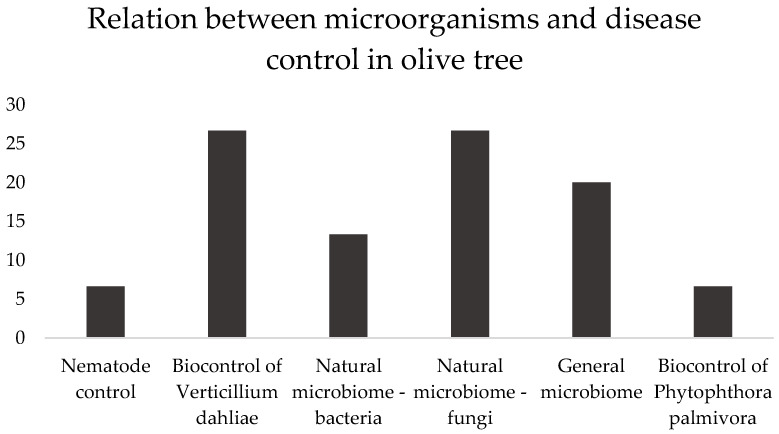
Subjects covered in the selected papers, in percentage, addressing the relation between microorganisms and disease control in olive tree, between brackets. Nematode control [51], Biocontrol of Verticillium dahlia [52,53,61,62], Natural microbiome—bacteria [54,59], Natural microbiome—fungi [55,56,57,58]. General microbiome [2,6,63], Biocontrol of Phytophthora palmivora [60].

In this section, the articles demonstrated six microbe actions: nematode control, biocontrol of *Verticillium dahliae* and *Phytophthora palmivora* (the main pathogens of olive trees worldwide), and the natural microbiome—bacteria or fungi or general (both), as presented in Figure 2.

For this purpose, Castillo et al. [51] investigated, under controlled greenhouse conditions in Barcelona (Spain), the performance of the AMF species Glomus intraradices, *Glomus mosseae*, or *Glomus viscosum* on the development of olive tree cvs. Arbequina and Picua, exposed to infection by nematodes *Meloidogyne incognita* and *Meloidogyne javanica*. The authors observed that AMF colonization increased vegetative growth by 88.9%, regardless of cultivar, age, and nematode infection, reducing the severity and reproduction of both Meloidogyne spp., evidencing the protective effect against parasitism. They concluded that pre-inoculation of olive trees with AMF can contribute to the healthy and vigorous growth of the tested cultivars during the seedling preparation phase, as already reported in previously presented studies.

Olive ancestors (wild plants) are considered sources of genetic variability that may contain important microorganisms with potential agronomic and biotechnological interest, particularly those antagonistic to *Verticillium dahliae*, the main pathogen of olive trees worldwide. Aranda et al. [52] developed a study to analyze the structure and diversity of bacterial communities in the rhizosphere and endosphere of wild olive trees from Cadiz and Cordoba, Spain. The results showed high heterogeneity in the composition of bacterial communities in the rhizosphere and suggested the existence of specific communities, with 14% of isolates having a high antagonistic activity against the pathogen. Among these, 58.5 to 78.3% showed proteolytic, lipolytic, and chitinolytic activity, with the production of indole-acetic acid (IAA) and siderophores, the majority belonging to the genus *Bacillus* (56.4%), *Pseudomonas* (27.7%), and *Paenibacillus* (7.4%). Thus, bacterial communities present in the rhizosphere and endosphere of wild olive trees may be promising as biocontrol agents against *V. dahliae* in olive trees and act as research possibilities in the sustainable management of the crop.

About 10 years ago, Schilirò et al. [53] commented that genetically based knowledge of interactions between beneficial bacteria and woody plants was highly limited and absent for olive trees. These authors studied the genetic responses (based on qRT-PCR and computational analysis by bioinformatics) of root colonization of olive tree cv. Arbequina by the native endophytic bacterium *Pseudomonas fluorescens* PICF7, an efficient biocontrol agent against the same Verticillium, an important root pathogen in olive cultivation. Computational analysis showed that the defense system and response to the provoked stresses represented nearly 45% of the genes induced through bacterial inoculation, inducing the biosynthesis of lipoxygenase, phenylpropanoids, terpenoids, and plant hormones. This was the first time that evidence was shown of the ability of an endophytic bacterium of the genus Pseudomonas spp. to trigger a series of defense responses when inoculated in an important woody species such as the olive tree, helping to explain its biocontrol activity.

To study and characterize the phyllosphere and carposphere (leaves and fruit), bacterial communities of mature olive grove cv. Maiatica (over 50 years old), subjected to 13 years of two different management types (sustainable versus conventional) in Ferrandina (southern Italy 40°29′ N, 16°28′ E), Pascazio et al. [54] used 16S rRNA and DGGE molecular methodologies. The authors observed that the olive tree presented the highest bacterial species number in the fruit under sustainable management. An improved understanding of the microbiota of the phyllosphere and carposphere of cultivated olive trees may be useful to promote plant growth, protection, and even improvement of crop quality.

Orazem, Celar, and Bohanec [55] first recorded the occurrence of fungi in olive micropropagation with a cold-tolerant cultivar, ‘Istrska belica’, in Slovenia. Fungi were isolated and identified, belonging to five genera: *Cladosporium*, *Chaetomium*, *Preussia*, *Biscogniauxia*, and *Sistotrema*, the last three isolated from the olive tree for the first time. Thus, the authors proved the presence of an endophytic fungal community in olive trees, potentially useful in studies concerning the control of the plant’s health.

Also evaluating the endophytic fungal community in different olive cultivars (Galega vulgar, Cobrançosa, and Olives), Materatski et al. [56] investigated the richness and diversity of fungi in different seasons and locations in the Alentejo region of Portugal. Variation was found among cultivars, seasons, and locations in the community investigated, of which cv. Galega vulgar appears as the richest and most diverse in the fall, and local Elva is the poorest and least diverse. The authors emphasized the importance of considering spatio-temporal distribution in endophytic fungal biodiversity in similar studies of olive cultivars.

Thus, continuing the interest in evaluating the endophytic fungal community in olive trees, Nicoletti, Vaio, and Cirillo [57] proposed a review, emphasizing its occurrence and the main effects of these microorganisms associated with olive trees. In the olive tree case, the increase in the number of studies on endophytic fungi in the past few years confirms that the spatio-temporal distribution of olive trees has been submitted to little investigation. However, the accumulated data provides evidence for understanding the impact of the role and use of this community as a biotechnology allied to the health of plants/cultivars.

Describing the microbiota in different olive cultivars may open new opportunities to understand how microbial interactions lead to the establishment or progression of diseases in olive trees. In this regard, limited knowledge exists about the endophytic microbiota in the phyllosphere (leaves and twigs) of olive trees, and this is considered a first step in understanding microbial interactions related to disease establishment. Costa et al. [58] evaluated, in mature olive groves (30–40 years old) of the Iberian Peninsula (Portugal and Spain), the endophytic fungal community of the phyllosphere of different olive cultivars (Cobrançosa, Galega vulgar, Madural, Picual, Verdeal Transmontana) using molecular methods (ITS1 barcode), revealing distinct communities of endophytic microorganisms, mainly yeasts, and also new organisms in all cultivars. Specifically, the cv. Galega exhibited a more diverse fungal community in contrast to cv. Picual, which exhibited a more diverse endophyte community compared to the others. Leaves tended to exhibit a more homogeneous community, while twigs differed more among cultivars, possibly due to higher irregularities in retaining microorganisms, unlike leaves. The authors reinforce that this difference between cultivars may explain the different susceptibility/tolerance to diseases recorded for olive trees. In particular, the cv. Cobrançosa, for example, has a great abundance of yeasts (mainly in the leaves), some with a recognized biocontrol action of anthracnose causes, for example.

Another important study was done by Fernández-González et al. [2], using root and soil samples collected from 36 olive cultivars of various origins at the World Olive Germplasm Collection (WOGC), (37°51′38.11″ N; 4°48′28.61″ W), located at the Institute of Agricultural and Fisheries Research and Training (IFAPA, Córdoba, Spain). The goal was to gather the first information on the microbiomes (communities of bacteria and fungi) of the endo- and rhizosphere. The results revealed that diversity in the endosphere was lower than in the rhizosphere, with a strong genotype influence of the cultivars. *Actinophytocola*, *Streptomyces*, and *Pseudonocardia* were the most abundant bacterial genera in the endorhizosphere, in contrast to Gp6, Gp4, *Rhizobium*, and *Sphingomonas* in the rhizosphere. *Canalisporium*, *Aspergillus*, Minimelanolocus, and *Macrophomina* were the major fungal genera of the root systems, but *most* of them were unidentified, indicating a potential that remains for future revisions by researchers.

Variation in bacterial communities living in the phyllosphere (epiphytically and endophytically) has established effects on host plants, although explanations are inconclusive or contradictory. Therefore, Mina et al. [59] evaluated bacterial communities on the surface and internally of the leaves and twigs of two olive cultivars (Cobrançosa and Verdeal-Transmontana), aiming to answer the previous question. Overall, Proteobacteria, Actinobacteria, and Firmicutes were the dominant phyla, with epiphytes being more diverse and abundant than endophytes. The genotype of the cultivar had the most marked effect on this result, especially for the epiphytic community, compared to the collection position effect (leaf versus twig).

To evaluate the biocontrol effect of mycorrhizal associations in the olive tree, Msairi et al. [60] set up an experiment under controlled conditions in Morocco involving AMF species and the pathogenic fungus *Phytophthora palmivora*. The AMF inoculum contained multiple species, including 22 species already identified in olive groves of the region. The AMF-colonized plants showed higher growth and were disease-free, even those inoculated with the pathogen, indicating that colonization led to resistance to *Phytophthora palmivora*. Inoculated plants showed 36 species of AMF and 121 spores/100 g of soil, different from those with the plant pathogen, showing 27 species and 67 spores/100 g of soil. The authors concluded that the olive tree is highly mycotrophic and forms an association stimulating not only area and root growth, but also a protective effect against root disease caused by *P. palmivora.* Thus, AMF introduction as a disease biocontrol agent can contribute to the development of more sustainable agriculture, reducing the application and negative effects of pesticides in olive cultivation, as already discussed.

Boutaj et al. [61] analyzed the efficiency of an AMF consortium in reducing symptoms caused by *Verticillium dahliae* in olive tree cv. Picholine Marocaine under controlled conditions. This consortium consisted of autochthonous fungi, obtained from five olive groves in Morocco, which presented 26 species. The AMF utilized were: *Claroideoglomus etunicatum*, *Rhizophagus prolifer*, *R. clarus*, *R. diaphanum*, *R. intraradices*, *Funneliformis mosseae*, *F. geosporum*, *Septoglomus constrictum*, *Diversispora versiformis*, *Glomus* sp1, *Glomus* sp2, *Glomus* sp3, *Glomus* sp4, *Glomus* sp5, *Acaulospora denticulata*, *A. spinosa*, *A. kentinensis*, *Acaulospora* sp1, *Acaulospora* sp2, *Acaulospora* sp3, *Acaulospora* sp4, *Entrophospora* sp1, *Gigaspora* sp1, *Gigaspora* sp2, *Gigaspora* sp3, and *Scutellospora* sp1. All were cultivated in pots with corn. The authors inoculated olive plants three months before contamination with the pathogen *Verticillium dahliae*, then they observed a high intensity of AMF colonization and a reduction of symptoms caused by the pathogen (dwarfism and leaf changes), which became more evident over time. The authors reinforced that the biostimulation caused by the consortium increased root colonization and, again, the tolerance/resistance of the plants to disease.

Thus, we know that different plant species, and even cultivars, promote different effects on associated microbial communities. In this regard, Fernández-González et al. [62] evaluated how fungal and bacterial communities vary in woody plants (two olive cultivars, tolerant and sensitive to the pathogen *Verticillium dahlia*) and wild oats (wild Halm oak), when cultivated in the same soil but under different management systems (agricultural versus native). Agricultural management of olive trees promoted differences in the microbiota when compared to natural soil, mainly with a higher abundance of the Gemmatinomonas and Fusarium genera, but with equal behavior of the cultivars against the target pathogen. In contrast to previously presented results, according to this result, the authors concluded that the composition and structure of rhizospheric communities under agricultural management have no differential role in the tolerance of olive cultivars to the pathogen *V. dahliae*.

The authors state that vascular pathogens are the main causative agents of diseases in olive trees, and the use of endophytic microorganisms is a promising strategy for controlling these diseases. Analyzing the structure and diversity of these microorganisms in the xylem of olive trees and characterizing this niche has been a challenge for researchers. An experiment conducted near Cordoba (southern Spain 37.5° N, 4.8° W, altitude 110 m), by Anguita-Maeso et al. [63], on almost adult olive seedlings of the cv. Picual and Arbequina (most planted cultivars in Spain) was proposed to characterize the chemical and microbial composition of xylem vessels. They used the spectroscopy techniques proton nuclear magnetic resonance (NMR) and inductively coupled plasma with optical emission spectroscopy (ICP-OES). The results showed a high concentration of sugars in the xylem vessels (54.35%), followed by alcohols (28.85%), amino acids (8.01%), organic acids (7.68%), and osmolytes (1.12%). Within these groups, the most notable were mannitol, ethanol, glutamine, acetic acid, trigonelline, K, and Cl, varying according to the age and genotype of the plant, with the levels of glucose, fructose, sucrose, mannitol, choline, B, and PO_4_^3−^ being higher in adult plants, and NO_3_^−^ with an opposite behavior, in both cultivars. In contrast, levels of aspartic acid, phenylalanine, and Na were higher in the cv. Picual than in Arbequina. The microbial composition was identified in the phyla Firmicutes (67%), Proteobacteria (22%), and Actinobacteriota (11%), with the most representative genera being *Anoxybacillus* (52%), *Cutibacterium* (7%), *Massilia* (6%), and *Pseudomonas* (3%). The authors concluded their study by stating that knowledge of the chemical composition of xylem vessels contributes to the improved understanding of the nutritional needs of microorganisms that inhabit this niche, including vascular pathogens and their antagonists, and may support the cultivation of these organisms under laboratory conditions. Thus, better technical facilities would be available to develop in vitro tests seeking sustainable solutions for the control of diseases in the olive crop.

A review of the past 10 years on beneficial microbial groups (AMF, RBPCP, plant growth promoting fungi, and other endophytes) in olive cultivation was carried out by Bizos et al. [6], addressing aspects of plant growth and productivity, biocontrol of pathogens, and amelioration of abiotic stresses. According to the authors, most studies have addressed the irregular *Rhizophagus* AMF and *Glomus mosseae*, and have proven increased root growth and improvements in the resistance of olive trees to environmental stresses and transplanting. Among the PGPR, the most notable were the species *Azospirillum* sp. and *Bacillus* sp., solubilizers of P and K, which promoted great growth of the olive tree when combined with other cultural practices. The endophytic bacteria *Pseudomonas fluorescens* and *Bacillus* sp., as well as fungal species of *Trichoderma* sp. were considered important biocontrol agents against olive diseases (such as *Verticillium* wilt, root rot, and anthracnose), and they are most efficient when present on the surface or inside the plant tissues in contact with the pathogens. Thus, the authors concluded that applying biocontrol agents in olive trees during the initial propagation stage is recommended because early treatment with bio-fungicides can significantly reduce the symptoms caused by pathogens in the field.

Table 2 contains a summary of the papers consulted involving the inoculation of microorganisms in olive trees with a view towards the biocontrol of diseases and pests.

### 3.3. Microbiology of Olive Trees in Brazil

Through the search for papers in scientific journals registered in the Scielo Brazil platform with the title “olive tree” or “Olea europaea” with no language limitation (the vast majority in Portuguese), we obtained between 50 and 60 results. The topics covered include, among others, leaf development, chemical composition or leaf nutrient contents, agronomic characterization of cultivars, in vitro germination/multiplication, cuttings/rooting (mostly), oil quality, agrometeorology, climate change, and extraction of leaf chemical compounds (oleuropein). A limited number of papers dealt with microbiology or microorganisms in olive trees/olive cultivation, indicating the extreme limitation of research and data with this focus, even with the positive and potential effects of using microorganisms in olive cultivation recorded in the international literature currently available.

**Figure 3:**
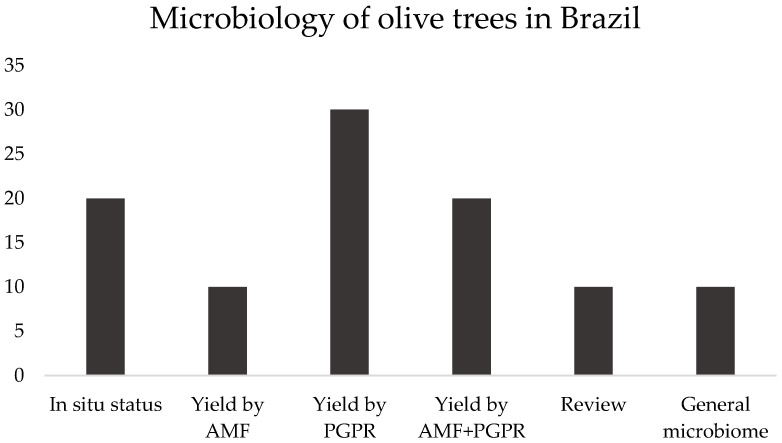
Subjects covered in the selected papers, in percentage, addressing the studies on microbiology of olive trees in Brazil, between brackets. In situ status [3], Yield by AMF [64], Yield by PGPR [65,66,69], Yield by AMF + PGPR [67,68], Review [5], General microbiome [70].

In this section, the articles involved six subjects: in situ status, yield promoted by AMF or PGPR or both, review, or general microbiome, as presented in Figure 3.

In 2015, the first study related to the use of AMF in olive seedling production appeared. Specifically, Ferreira et al. [64] evaluated the effect of using different AMF species in the growth of seedlings of different olive cultivars with potential cultivation in the southern region of Minas Gerais. The experiment was carried out under controlled conditions, evaluating three olive cultivars (Arbequina, Grappolo 541 (MGS GRAP541), and Maria da Fé (MGS MARIENSE) and four inoculation treatments with AMF (no inoculum and AMF Glomus clarum, Gigaspora rosea, or Acaulospora scrobiculata). The studied AMF group provided higher shoot and root dry matter mass of the seedlings when compared to the non-inoculated seedlings, especially for Grappolo 541 (MGS GRAP 541) and Arbequina. The cultivar Maria da Fé (MGS MARIENSE) showed low mycorrhizal dependence and lower shoot dry matter production compared to the other cultivars.

In 2017 new studies emerged employing PGPR inoculation under controlled conditions. Thus, Silva et al. [65] evaluated the effect of PGPR inoculation on the rooting of cuttings of olive tree cv. Ascolano 315, Arbequina, and Grappolo 541 under controlled conditions in the location Maria da Fé (MG, Brazil). The isolated rhizobacteria from the Biological Reserve Serra dos Toledos (Itajubá-MG) were phenotypically characterized under different growth conditions, before inoculation and comparison of the effect of indole-butyric acid (IBA, a commercial treatment). Some isolates showed phosphate solubilization ability, growing in wide ranges of pH (5.0 to 9.0) and temperature (15–35 °C), with EIA production ranging from 110.53 to 383.58 µg mL^−1^. All isolates showed different rooting potentials of olive tree cuttings, some with performances similar to those provided by AIB. Thus, this study proved that rhizobacteria with EIA production should be commercially tested in the production of olive seedlings in Brazil, and can be the research target of future studies.

Therefore, Mariosa et al. [66] evaluated the potential of rhizobacteria (isolates 32, 39, 42, and 48) and the type-strains *Azospirillum brasilense* (BR 11001^t^), *Azospirillum amazonense* (BR 11040^t^), *Herbaspirillum seropedicae* (BR 11175^t^), and *Burkholderia brasilensis* (BR 11340^t^) to promote the rooting and growth of olive seedlings from semiligneous cuttings of cv. Grappolo 541. The isolates and type strains showed in vitro indoleacetic acid (IAA) production ranging from 200 to 1406 μg mL^−1^, with isolates 39 and 48 being the highest producers. However, we found no correlation between IAA production obtained in vitro and rooting. The strain-type *H. seropedicae* (BR 11175^t^), due to its root growth incentive, presented a potential for use in the rooting and growth of olive tree seedlings of the employed cultivars, compared to the hormone indolebutyric acid (IAB), commercially used in seedling formation.

The effect of AMF and PGPR co-inoculation on olive seedling growth was seen for the first time in 2019 and has scarcely been explored in Brazil. For this purpose, Costa and Melloni [67], under controlled greenhouse conditions, evaluated, in the cultivars Arbequina and Maria da Fé, the role of cross-inoculation of the rhizobacteria *Pseudomonas* sp, *Paenibacillus* sp1, and *Paenibacillus* sp2, and the AMF group *Acaulospora scrobiculata*, *Gigaspora rosea*, and *Rhizophagus clarus* on the growth and formation of AMF symbiosis. Each cultivar showed different results, and for some attributes, the isolated effect of AMF or the co-inoculation of AMF and rhizobacteria significantly increased the growth of olive tree seedlings.

In Brazil, the most frequently used method for the propagation and production of olive tree seedlings is cuttings, although the rooting rate is limited for some cultivars. In this regard, Ritter et al. [68] evaluated the use of AMF and plant growth-promoting rhizobacteria (PGPR) in the rooting of olive tree cuttings in the municipality of Marechal Cândido Rondon (PR), at the coordinates of latitude 24°32′22″ S, longitude 54°03′24″ W, and 420 m in altitude. The authors tested cuttings from four cultivars (Ascolano 315, Koroneiki, Maria da Fé, and Picual) of four-year-old plants, which received inoculation with three species of AMF (*Gigaspora margarita*, *Glomus clarum*, and *Glomus etunicatum*). These were compared with three concentrations of commercial inoculant containing *Azospirillum brasilense* (0, 25 mL L^−1^, 50 mL L^−1^ and 75 mL L^−1^ of Nitro1000^®^ in water), after a treatment with 3000 mg L^−1^ of indolebutyric acid (IAB). After 75 days, the authors found that the use of AMF in the cultivars Maria da Fé and Picual affected rooting positively, including leaf number and root length. Only the cvs. Maria da Fé and Koroneiki responded to inoculation with *A. brasiliense*, with better rooting percentages using 75 and 52 mL L^−1^, respectively. Thus, the authors concluded that the use of microorganisms proved its efficiency in seedling formation, despite the high diversity of cultivars, AMF, and PGPR, which also requires studies for its expanded application in olive cultivation.

Thus, a new study with the objective of evaluating the capacity of different AMF species (*Rhizophagus clarus*, *Gigaspora rosea*, or *Acaulospora scrobiculata*), combined or not with IBA or rhizobacteria, was conducted to promote the rooting of cuttings of three olive tree cultivars (Arbequina, Grappolo 541, and Maria da Fé), in Maria da Fé, Brazil [69]. The methodology involved olive tree cuttings inoculated with (I) AMF, (II) AMF combined with increasing doses of IBA, and (III) AMF combined with three isolates of rhizobacteria. The inoculation of olive tree cuttings of cultivars Arbequina, Grappolo 541, and Maria da Fé with AMF, singly or combined with IBA or rhizobacteria did not significantly promote rooting. In this sense, alternative forms of promoting rooting of olive tree cuttings are still a challenge, and further studies for standardizing methodologies and experimental conditions are required.

Of the aforementioned works involving the effect of the inoculation of microorganisms on the development of olive tree cuttings, in Brazil, a summary can be seen in Table 3.

Costa, Melloni, and Ferreira [5] proposed a review of the biotechnological potential of certain soil microorganisms, particularly AMF and rhizobacteria. The authors found that several studies have been conducted abroad addressing such microbial groups in olive trees, with significant results regarding the growth and development of co-inoculated plants, and improvements in resistance to heavy metals and pathogens, both under nursery and field conditions. However, in Brazil, despite the great potential for olive farming, mainly due to its territorial extension, and climatic and biological diversity, we find a gap of knowledge in this area, impeding the comprehension and the increase of olive cultivation in the country.

PGPR can provide improvements in plant development through a variety of mechanisms. One such mechanism deals with producing plant growth regulators, such as IAA. Ramos et al. [70] evaluated the IAA production potential of rhizobacteria obtained from the rhizosphere of 17 different olive cultivars from the germplasm bank of the Agriculture and Crops Research Company of Minas Gerais (EPAMIG), in Maria da Fé, MG (Brazil). The authors observed a high phenotypic similarity among isolates (93% to 100%), and relevant indexes of IAA production (0.16 and 29.08 μg mL^−1^). At the end of their study, the authors indicated isolates with potential for use in future trials in the induction of rooting of olive tree cuttings.

In the same germplasm base, Melloni et al. [3] evaluated the occurrence of AMF symbiosis and propagules in the rhizosphere of 17 cultivars of national importance. The root colonization in situ was between 0.4% and 3.6%, with no influence of cultivar on the total spore number and AMF diversity; the species *Glomus ambisporum* and *Acaulospora scrobiculata* were found the most frequently in the rhizospheres of the olive trees analyzed. These data reinforced olive mycotrophism, despite low colonization due to inefficiencies of native soil fungal communities and/or application of management practices with negative effects on the establishment of symbiosis (e.g., fertilization and vegetation cover control).

Finally, an unprecedented study of the microbiome was conducted in Brazilian olive groves in 2022. Specifically, the diversity and potential interactions of endophytic (leaf) bacteria and fungi were analyzed in different (unidentified) olive cultivars by Oliveira et al. [71] using culture-dependent and culture-independent methods (16S rRNA and ITS1). Specifically, 93 plants were sampled in nine locations across the country, including the states of São Paulo and Minas Gerais. For the bacterial community, the most frequent genera were Stenotrophomonas and Achromobacter. Regarding fungi, a total of 800 isolates was obtained, grouped in 38 genera, with 32 of them never previously described in Brazilian olive trees. The most frequent fungal genera were *Pseudocercospora*, *Hyphozyma*, and *Symmetrospora*, with the most abundant trophic level consisting of the pathotrophs, followed by the symbiotrophs. The authors revealed that the endophytic microbial community varied with plant age, altitudinal gradient (except for bacteria), and geographic location, with no effect on the olive cultivar.

### 3.4. Future Perspectives

Through analysis of the results of this review, we can say that the management variations applied in olive cultivation around the world tend to reinforce the importance of investing in more sustainable technologies when aiming to conserve or increase the quality/functionality of soils. In this sense, and based on the previous statements, we can assure that the ideal olive cultivation would be that which emphasizes the maintenance of soil cover with spontaneous plants, limits use of agrochemicals in the control of plants and diseases or pests, employs a light pruning system, and with residues retained in the cultivation area. The use of organic conditioners derived from the industrial processes of olive oil production, the inoculation of microorganisms mainly in the seedling production phase to minimize biotic and abiotic stresses, and the use of cultivars genetically adapted to specific areas and climatic conditions for their development and production are proposed as equal measures.

Brazilian researchers are encouraged to incorporate into research, under controlled and field conditions, the current knowledge found in the international literature on sustainable management applied by the world’s largest olive oil researchers or producers, mainly regarding microorganisms and their processes, and adapting them, when necessary, to the soil and climatic conditions in Brazil. Only thereafter would the research gaps previously presented be (re)worked, and new biotechnological advances could contribute to olive cultivation success in the country.

## Figures and Tables

**Table 1:** Effects of microorganism inoculation on yield, absorption of nutrients, soil conditions, and resistance to drought in olive tree.

Paper	Microorganisms Inoculation	Main Results
[32]	*G. mosseae*, *G. intraradices* or *G. claroideum*	Decrease of salinity effects (*G. mosseae*) and increase of K absorption and root and shoot development
[34]	*Rhizophagus irregularis* (*G. intraradices*)	Decrease of transplanting and drought stresses
[37]	*G. intraradices*	Changes in composition of sugars and bacteria in rhizosphere
[38]	*Gigaspora* sp. or *Glomus* sp.	Increase of shoot development and leaf absorption of Mn, Fe, Zn, Ca, Mg, K, and P
[39,40]	AMF commercial	Increase of P and decrease of Mn absorption
[41]	*R. irregularis*, *Septoglomus deserticola* and *G. gigantea*	Higher aggregate stability
[42]	6 AMF: *Glomus etunicatum*, *Glomus microaggregatum*, *Glomus intraradices*, *Glomus claroideum*, *Glomus mosseae* and *Glomus geosporum*	Decrease of drought stresses, increase of K, N, Zn, and Fe absorption
[44]	9 AMF: 7 of the genus *Glomus*, and 2 of the species *Rhizophagus irregularis* and *Paraglomus brasilianum*.	Decrease of drought stresses and increase of plant growth
[45]	*G. deserticola* and/or *G. margarita*	Decrease of drought stresses
[46]	*G. deserticola* or *G. margarita* or both	Decrease of Na and Cl absorption and increase of macro and micronutrients
[48]	*R. irregularis*	Decrease of drought stresses

**Table 2:** Effects of microorganism inoculation on biocontrol of diseases and pests in olive trees.

Paper	Microorganism Inoculation	Main Results
[6]	Review—AMF: *Rhizophagus* e *G. mosseae*; PGPR: *Azospirillum* sp. and *Bacillus* sp.; and *Trichoderma* sp.	Biocontrol of many diseases (*Verticillium*, anthracnose, etc.)
[51]	AMF: *Glomus intraradices*, *Glomus mosseae* or *G. viscosum*	Reduction of infection by *Meloidogyne* sp. (nematodes).
[53]	*Pseudomonas fluorescens*	Biocontrol of *Verticillium dahliae*
[60]	AMF: 22 species	Biocontrol of *Phytophthora palmivora*
[61]	AMF: 26 species	Biocontrol of *Verticillium dahliae*

**Table 3:** Effects of microorganism inoculation in olive tree cuttings cultivated in Brazil.

Paper	Microorganism Inoculation	Main Results
[65]	AMF: *Glomus clarum*, *Gigaspora rosea* or* Acaulospora scrobiculata*	Increase of shoot and root dry matter mass of Grappolo and Arbequina cultivars
[66]	Many isolates of PGPR (not identified)	Diverse effects on rooting of cuttings
[67]	Isolates of PGPR and *Azospirillum brasilense*, *Azospirillum amazonense*, *Herbaspirillum seropedicae* and *Burkholderia brasilensis*	Diverse IAA producers caused an increase of rooting with *H. seropedicae*
[68]	Isolates of PGPR (*Pseudomonas* sp, *Paenibacillus* sp1, and *Paenibacillus* sp2), and AMF (*Acaulospora scrobiculata*, *Gigaspora rosea*, and *Rhizophagus clarus*)	Increase of shoot dry matter mass of cultivars
[69]	AMF: *G. margarita*, *G. clarum* or *G. etunicatum* and PGPR *A. brasilense*	Increase of rooting, leaf number and root length of Maria da Fé and Picual cultivars
[70]	AMF: *R. clarus*, *G. rosea* or *A. scrobiculata* + PBA + PGPR *Paenibacillus polymyxa* and *Pseudomonas protegens*	No effects on rooting of cuttings

## Data Availability

Not applicable.
